# Melatonin Improves the Quality of Inferior Bovine Oocytes and Promoted Their Subsequent IVF Embryo Development: Mechanisms and Results

**DOI:** 10.3390/molecules22122059

**Published:** 2017-11-27

**Authors:** Minghui Yang, Jingli Tao, Menglong Chai, Hao Wu, Jing Wang, Guangdong Li, Changjiu He, Lu Xie, Pengyun Ji, Yunping Dai, Liguo Yang, Guoshi Liu

**Affiliations:** 1 National Engineering Laboratory for Animal Breeding, Key Laboratory of Animal Genetics and Breeding of the Ministry of Agriculture, Beijing Key Laboratory for Animal Genetic Improvement, College of Animal Science and Technology, China Agricultural University, Beijing 100193, China; Yangmh16@cau.edu.cn (M.Y.); Taojl16@cau.edu.cn (J.T.); cml313@163.com (M.C.); 18800160525@163.com (H.W.); caylajingjing@gmail.com (J.W.); 15600911225@cau.edu.cn (G.L.); chungjoe@mail.hzau.edu.cn (C.H.); luxiecau@163.com (L.X.); jipengyun1989@126.com (P.J.); 2 College of Animal Science and Technology, Huazhong Agricultural University, Wuhan 430070, China; yangliguo2006@foxmail.com; 3 College of Biological Sciences, China Agricultural University, Beijing 100193, China; Daiyunping@sina.com

**Keywords:** melatonin, inferior oocytes maturation, mitochondria, in vitro embryo production

## Abstract

The inferior oocytes (IOs), which are not suitable for embryo development, occupy roughly one-third or more of the collected immature bovine oocytes. The IOs are usually discarded from the in vitro bovine embryo production process. Improving the quality of the inferior oocytes (IOs) and make them available in in vitro embryo production would have important biological, as well as commercial, value. This study was designed to investigate whether melatonin could improve the quality of IOs and make them usable in the in vitro maturation (IVM) and subsequent (in vitro fertilization) IVF embryo development. The results indicated that: the maturation rate of IOs and their subsequent IVF embryo developments were impaired compared to cumulus-oocyte complexes and melatonin treatment significantly improved the quality of IOs, as well as their IVF and embryo developments. The potential mechanisms are that: (1) melatonin reduced reactive oxygen species (ROS) and enhanced glutathione (GSH) levels in the IOs, thereby protecting them from oxidative stress; (2) melatonin improved mitochondrial normal distribution and function to increase ATP level in IOs; and (3) melatonin upregulated the expression of *ATPase 6*, *BMP-15*, *GDF-9*, *SOD-1*, *Gpx-4*, and *Bcl-2*, which are critical genes for oocyte maturation and embryo development and downregulated apoptotic gene expression of caspase-3.

## 1. Introduction

The increasing importance of in vitro production of bovine embryos in commercial cattle breeding programs demands an improvement of embryo viability [1]. Oocytes in vitro maturation is one of the most important steps in the in vitro embryo production process, and it is crucial for the success of this process [2,3]. In many laboratories, almost half of collected cumulus-oocyte complexes (COCs) were discarded due to their poor quality. These oocytes are classified as inferior oocytes (IOs). Researchers have tried intracytoplasmic sperm injection to improve the embryo production efficiency of the IOs [4]. Although, this was a viable alternative to increase IVF embryo production of IOs, it is still hard to popularize it in commercial production. It is the intrinsic quality of the oocyte that determines the proportion of oocytes developing to blastocysts; however, the culture environment has a critical impact on the development of embryos [5,6,7]. As a result, the most effective method to reverse the fate of IOs should be an improvement of their in vitro developmental environments. 

Oxidative stress causes poor quality of the oocytes [5] and it is a major culprit responsible for low efficiency in oocyte maturation and embryo development in several species [6]. The role of reactive oxygen species (ROS) and antioxidants in relation to female reproductive function has been a subject of recent research interest [7,8]. Oxidative stress negatively impacts oocyte maturation and antioxidants protect oocytes from oxidative stress [9,10]. For example, the antioxidant glutathione (GSH) plays an important role in the antioxidant system of cells [11]. The concentrations of GSH in matured oocytes are significantly higher than that in the immature oocytes and play an important role for the successful fertilization [12]. 

Melatonin is another potent and naturally-occurring antioxidant [13]. It is produced not only in the pineal gland, but also in skin [14], neuronal cell [15], and in oocytes [16]. It improves oocyte quality and fertilization rates [17]. Several studies have shown that the presence of melatonin in the follicular fluid and its levels positively correlates with oocyte quality and maturation [18,19,20,21]. In human ovarian follicular fluid, melatonin’s concentration was three-fold higher than in serum [19]. The concentration of melatonin was higher in the fluid of large follicles than in the fluid of small follicles in patients undergoing IVF-embryo transfer [22]. Melatonin was also detected in porcine follicular fluid. All of these suggest that melatonin is directly involved in ovarian function in mammals [23]. It was reported that melatonin administration improved oocyte quality [19] and melatonin supplementation during IVM of COCs resulted in a greater proportion of oocytes which extruded the polar body with relatively low ROS levels in porcine [24] and in bovine [25] specimens. Oxidative stress also causes the abnormal mitochondrial distribution and damage in IOs. Melatonin is also a mitochondrial-targeted antioxidant and it protects the mitochondria by scavenging reactive oxygen species (ROS), inhibiting the mitochondrial permeability transition pore (MPTP), and activating uncoupling proteins (UCPs) [16,26]. In the current study, the effect of melatonin on mitochondrial distribution and functions in IOS is also investigated.

The classification of IOs was based on surrounding cumulus cell layers and homogeneity of ooplasm. The IOs then were selected as a target cells to study the effects and mechanisms of whether, and how, melatonin improves their quality and maturation. The address was given to the effects of melatonin at the concentration of 10^−9^ M, which is considered as physiological concentration, on ROS, GSH, oocyte mitochondrial distribution, and function in in vitro-matured IOs. In addition, the oocyte maturation-associated genes, as well as their effects on the in vitro production of bovine embryos, were also investigated.

## 2. Results

### 2.1. The Gene Expression Level of Pro-Apoptotic Genes Caspase-3, -9, and Bax (Relative mRNA) in IOs and COCs

**Figure 1:**
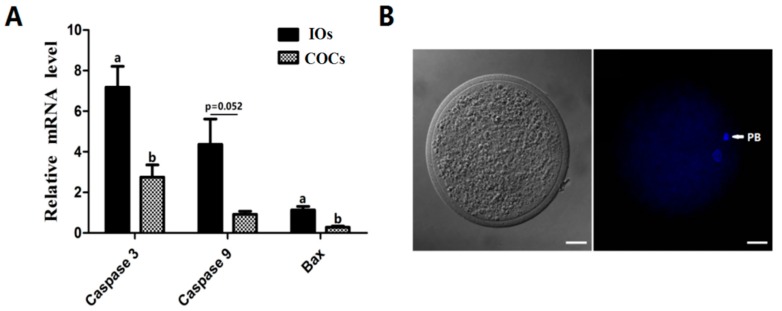
The relative mRNA expression of *caspase-3*, *-9*, and Bax in IOs and COCs. (**A**) Relative mRNA expression of *caspase-3*, *-9*, and *Bax* in IOs and COCs. IOs: inferior oocytes; COCs: cumulus-oocyte complexes. *n* = 3. ^a,b^ Values of different superscripts indicate significant difference (*p* < 0.05). (**B**) Nuclear staining after IVM. PB: polar body, scale bar = 20 μm.

The results showed that expressions of pro-apoptosis related gene *caspase-3* and *Bax* in IOs were significantly higher than that in COCs (*p* < 0.05), The expression of *caspase-9* in IOs was also higher than that in COCs even it failed to reach the significant difference (*p* = 0.052) (Figure 1A).

### 2.2. The Effect of Melatonin on the Nuclear Maturation of Bovine IOs

As shown in Table 1, the MII rate of the IOs + MT (10^−9^ M) group (71.4 ± 1.88%) was significantly higher than that in IOs group(59.4 ± 3.14%; *p* < 0.05); however their MII rates were still significantly lower than that in COCs group (87.9 ± 0.64%; *p* < 0.01).

### 2.3. The Effects of Melatonin on the ROS and GSH Levels in MII Oocytes

**Figure 2:**
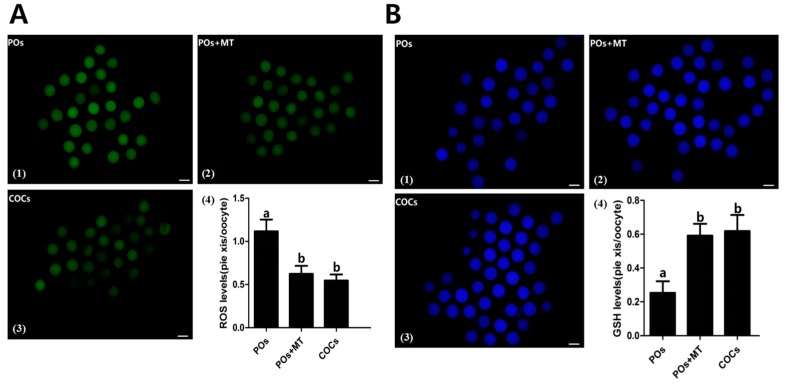
Effects of melatonin on ROS as well as GSH levels in bovine IOs. (**A**) Effects of melatonin on levels of ROS in IOs; A(1–3) the representative images of the H2DCFDA fluorescence staining. The higher green intensity indicated more ROS; scale bar = 100 μm; A(4): the statistical analysis of the data from A(1–3); *n* = 4; (**B**) Effects of melatonin on levels of GSH in IOs, B(1–3) the representative images of the GSH fluorescence staining. The higher the blue intensity is the more the GSH; scale bar = 100 μm; B(4) the statistical analysis of the data from B(1–3); *n* = 4. ^(a,b)^ Values of different superscripts indicate significant difference (*p* < 0.05).

The results showed that the levels of ROS were significantly lower in melatonin-treated IOs oocytes (0.62 ± 0.093) than that in IOs (1.12 ± 0.136) (*p* < 0.05, Figure 2A). Interestingly, melatonin (10^−9^ M) treatment led the ROS of IOs to reduce to the similar levels of the COCs control (0.55 ± 0.070), *p* > 0.05. In contrast, the level of GSH was significantly higher in melatonin treated IOs than that in IOs (0.59 ± 0.069 vs. 0.25 ± 0.069, *p* < 0.05 Figure 2B).

### 2.4. Effects of Melatonin on the Function of Mitochondria

**Figure 3:**
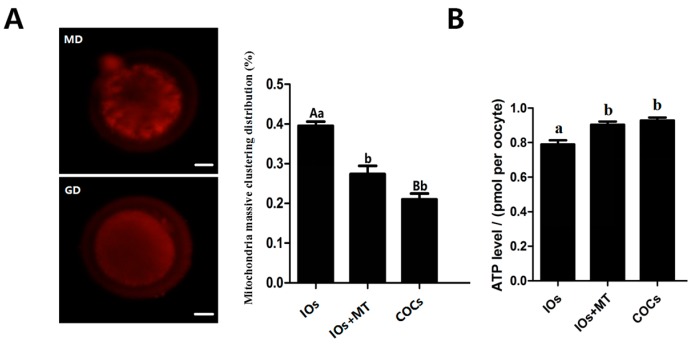
Effects of melatonin on mitochondria distribution and ATP production in MII-stage oocytes. (**A**) The state of mitochondria distribution, (mean ± SEM of 87 oocytes). The red fluorescence represents mitochondria. MD: the representative image of mitochondrial massive clustering distribution. GD: the representative image of mitochondrial granulated distribution; scale bar = 20 μm; the bar graph was the statistical analysis of the mitochondrial distribution in oocytes; (**B**) Cytoplasmic ATP levels in individual MII bovine oocytes (mean ± SEM of 85 oocytes). ^a,b^ Values of different superscripts indicate significant difference (*p* < 0.05); ^A,B^ Values of different superscripts indicate highly significant difference (*p* < 0.01).

The results indicated that melatonin (10^−9^ M) treatment significantly reduced the massive clustering distribution rate of mitochondria compared to the non-treated MII-stage IOs (0.40 ± 0.011 vs. 0.27 ± 0.021, *p* < 0.05); There were no significant differences were observed between melatonin-treated oocytes with COCs control (0.27 ± 0.021 vs. 0.21 ± 0.014, *p* > 0.05, Figure 3A). The ATP level in melatonin-treated IOs oocytes was also higher than that in non-treated IOs (0.90 ± 0.018 vs. 0.79 ± 0.024 pmol/per oocyte, *p* < 0.05) and it was similar to the ATP level of COCs of controls (0.90 ± 0.018 vs. 0.93 ± 0.017 pmol/per oocyte, *p* > 0.05, Figure 3B).

### 2.5. Effect of Melatonin on Expression of HSP90 in Bovine IOs

**Figure 4:**
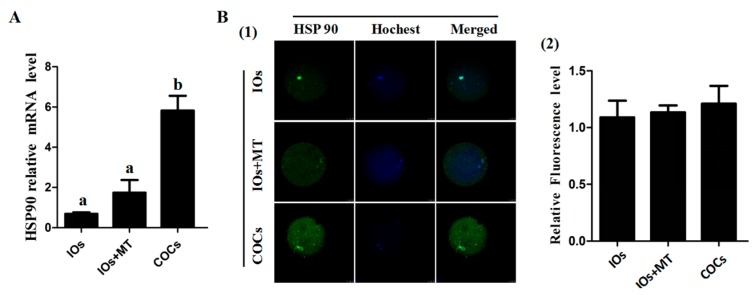
Effects of melatonin on expression of HSP90 in bovine oocytes. (**A**) Effects of melatonin on the levels of HSP90 mRNA; (**B**) the effects of melatonin on the HSP90 protein levels. B(1) The representatives of immunofluorescent inmages of HSP90 protein in MII bovine oocytes, scale bar = 25 μm; and B(2) The statistical analysis of the data from B1, HSP90 fluorescence intensity was analyzed using in MII-stage oocytes (mean ± SEM of 42 oocytes). ^a,b^ Values of different superscripts indicate significant difference (*p* < 0.05).

As shown in Figure 4, the *HSP90* mRNA expression in IOs was significantly lower than that in COCs control group (*p* < 0.05), and It appeared that melatonin (10^−9^ M ) treatment up-regulated the *HSP90* mRNA level in IOs but this upregulation failed to achieve a significant difference. In contrast, HSP90 protein expression showed no significant difference among the groups and this indicated post transcriptional regulation for HSP90 in oocytes.

### 2.6. The Effect of Melatonin on Expressions of GPX-4, SOD-1, and Bcl-2

**Figure 5:**
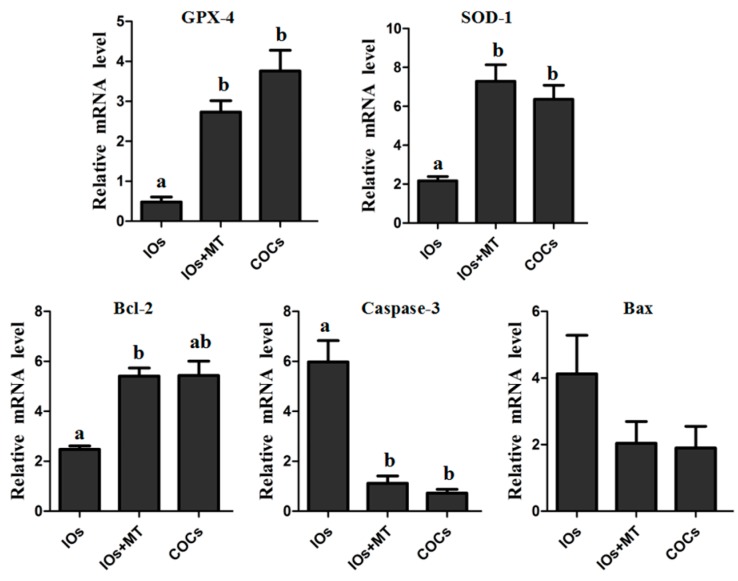
Relative expression levels of *GPX-4*, *SOD-1*, *Bcl-2*, *Caspase-3* and *Bax* in MII oocytes. ^a,b^ Values of different superscripts indicate significant difference within the expression level of each gene (*p* < 0.05). COCs, cumulus-oocyte complexes; IOs, inferior bovine oocytes; MT, melatonin.

The gene expressions of *GPX-4* and *SOD-1* which are anti-oxidative genes were significantly up-regulated by melatonin (10^−9^ M) treatment in IOs (*p* < 0.05, Figure 5), and their levels after melatonin treatment were similar to the expression level of COCs group (*p* > 0.05). Melatonin also significantly upregulated the expression of anti-apoptotic gene *Bcl-2* in IOs (*p* < 0.05), In contrast, the gene expression of pro-apoptosis related gene *caspase-3* was downregulated by melatonin treatment in IOs compared to its untreated counterparts (*p* < 0.05). The expression of *Bax*, however, was not significantly influenced by melatonin treatment (*p* > 0.05).

### 2.7. The Effect of Melatonin on Expression of Oocytes Maturation-Related Genes (GDF-9, BMP-15, ATPase 6, and ATPase 8)

**Figure 6:**
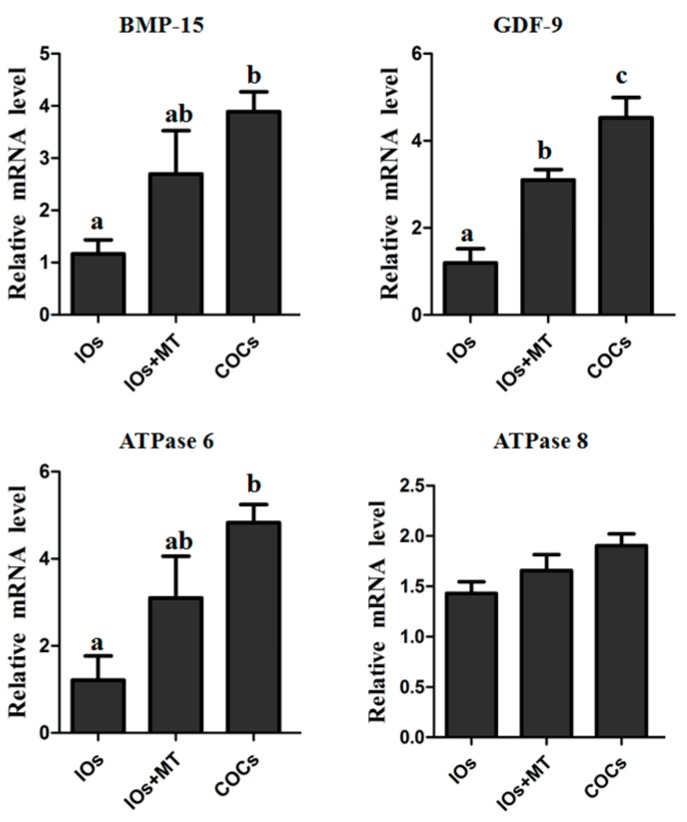
Relative expression levels of BMP-15, GDF-9, ATPase 6 and ATPase 8 in MII oocytes. ^a–c^ Values of different superscripts indicate significant difference within the expression level of each gene (*p* < 0.05). COCs, cumulus–oocyte complexes; IOs, inferior bovine oocytes; MT, melatonin.

The relative expression levels of *GDF-9* mRNA in MII oocytes in the IOs + MT (10^−9^ M) group was significantly higher than that in non-treated IOs (*p* < 0.05, Figure 6), but still lower than that in COCs control group (*p* < 0.05). The relative mRNA expression levels of *BMP-15* and *ATPase 6* in the IOs group was significantly lower than that in COCs control group (*p* < 0.05). Melatonin supplement upregulated their expression compared to the non-treated IOs. There were no significant difference as to the gene expression of *ATPase 8* among the groups (*p* > 0.05, Figure 6).

### 2.8. The Effects of Melatonin on IVF Embryo Developmental Potential and Cell Number of Blastocyst Obtained from IOs

**Figure 7:**
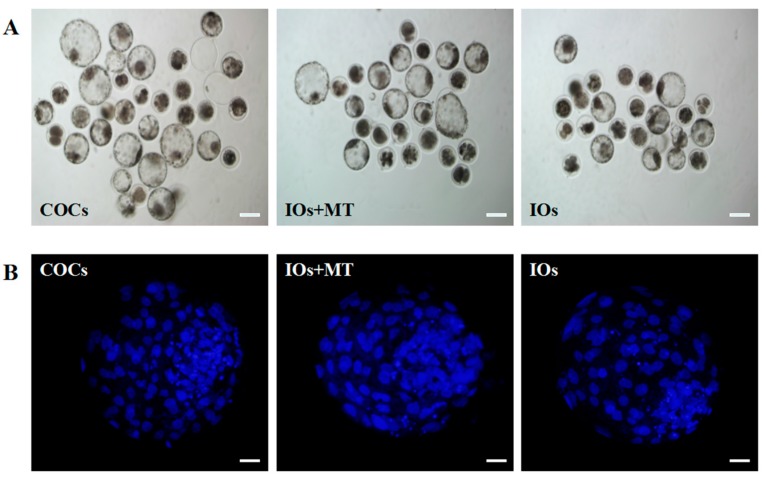
Effects of melatonin on IVF embryo developmental potential and cell number of blastocysts. (**A**) Epifluorescence photomicrographs of in vitro-produced bovine blastocysts. COCs, cumulus–oocyte complexes; IOs, inferior bovine oocytes; MT, melatonin; scale bar = 100 μm; (**B**) Nuclear staining of bovine blastocyst after IVF in different groups. Scale bar = 20 μm.

As shown in Table 2, the cleavage rates of the IOs were significantly lower than that of COCs (66.1 ± 2.64 vs. 90.5 ± 0.60%, *p* < 0.01), and melatonin(10^−9^ M ) supplement improved the cleavage rates of IOs to 79.8 ± 2.42% which was significantly higher than that in IOs alone (*p* < 0.05), the similar results were observed in the blastocyst rate. The blastocyst rates of the IOs was significantly lower than that of COCs (33.1 ± 0.87% vs. 44.0 ± 0.74%, *p* < 0.01), and melatonin (10^−9^ M) supplement significantly improved the blastocyst rates of IOs to 38.5 ± 1.11%, (38.5 ± 1.11% vs. 33.1 ± 0.87%, *p* < 0.05, Table 2). The data also showed that the blastocyst cell number in IOs significantly lower than that of COCs control (104.89 ± 3.51 vs. 122.33 ± 3.57, *p* < 0.01), and melatonin (10^−9^ M) treatment increased the blastocyst cell number of IOs to 115.78 ± 1.714, which was significantly higher than that of IOs alone (104.89 ± 3.51, *p* < 0.05. Figure 7, Table 3).

## 3. Discussion

In the current study, the effects of melatonin on improving the quality of bovine IOs during their maturation have been systematically investigated. Previous studies reported that melatonin scavenged ROS and promoted the in vitro maturation rate of oocytes in different species including human [19], porcine [20,24], mice [16], and bovine [21,25] models. However, these studies were performed by use of oocytes with good quality and little has been known to the effect of melatonin on the IOs which were usually discarded from the studies. Identification of the effective methods to improve the quality of these IOs has significant biological as well as commercial prospects regarding the in vitro embryo production.

One of the obstacles for in vitro oocyte maturation is its excessively-produced ROS. This ROS jeopardizes the quality of oocytes and, therefore, hinders the oocyte’s maturation and causes their apoptosis [16]. In this study, we found that the bovine IOs had a relatively high level of ROS and low level of GSH compared to normal COCs. This might attribute to a poor efficiency of IOs for their in vitro maturation (Figure 2A). To combat the negative effects of the excessive ROS and promote the oocyte’s maturation, the antioxidants are frequently used in the in vitro culture system [27,28]. In this study, a potent naturally-occurring antioxidant melatonin was selected. Melatonin is present in follicular fluids in relatively high levels [18,19,22], partially because oocytes have the capacity to synthesize melatonin per se [29]. It was found that melatonin added into the culture medium significantly reduced the ROS and increased GSH level of the oocytes (Figure 2). Melatonin directly scavenges the ROS. In addition, it also upregulates mRNA levels of antioxidant enzymes. Antolin et al. [30] reported that melatonin enhanced mRNA levels of both Cu,Zn-SOD and Mn-SOD in cells. Mayo et al. [31] investigated the mechanisms by which melatonin upregulated the antioxidant enzyme gene expression; they found that melatonin-induced synthesis of new proteins of all the three antioxidant enzymes, i.e., Cu,Zn-SOD, Mn-SOD, and GSH-Px. Our study confirmed that melatonin upregulated the gene expressions of several antioxidant enzymes (Figure 5).

Melatonin not only reduced ROS level but also improved mitochondrial function (Figure 3). Mitochondria are a major source of ROS production and they require additional protection from oxidative stress [32,33]. Previous studies have demonstrated that melatonin preserves the optimal mitochondrial function and homeostasis by reducing mitochondria oxidative stress [34,35,36]. It is recognized that mitochondria distribution is a dynamic process [37], and it is an important indicator of oocyte quality. For example, a uniform, granulated distribution of active mitochondria in the process of oocyte maturation and also in the early embryo specific period is essential for the normal embryo development [38,39,40]. Both the mitochondria content and ATP levels are positively associated with the developmental competence of oocytes, that is, they promote the cytoplasmic maturation of oocytes and their IVF embryos’ development [41,42]. In the current study, we observed that, in IOs, the mitochondria were clustered to one side of the oocytes and melatonin treatment significantly normalized the mitochondrial distribution and improved their function to produce more ATP (Figure 3). This phenomenon has never been reported previously.

Another important factor related to the quality of oocytes is HSP90 expression. HSP90 is regarded as a molecular chaperone. It plays a crucial role in the folding, transporting, and assembling proteins and, furthermore, it protects the cell under different stress conditions and inhibits apoptosis through several mechanisms [43,44,45]. Studies showed that the HSP90 level in bull spermatozoa gradually declined following the process of freezing-thawing, and might be associated with sperm plasma membrane integrity and acrosome integrity [46]. HSP90 was able to repair chromosome damage caused by freeze-thawing, and maintain DNA integrity [47]. Melatonin was reported to turn on the production of HSP90 [48,49]. However, the exact relationship between melatonin and HSP90 on immature oocytes is essentially unknown. In this study, it was observed that HSP90 mRNA expression in IOs was significantly lower than that in COCs (*p* < 0.05); however, melatonin treatment failed to upregulate HSP90 mRNA expression (Figure 4). It appeared that HSP90 was not involved in the pathway in which melatonin improved the quality of bovine IOs.

**Figure 8:**
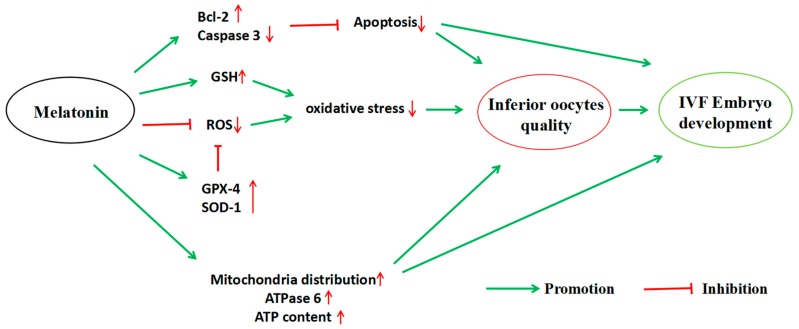
The action pathway connecting the beneficial effect of melatonin on bovine inferior oocytes and their subsequent IVF embryo development.

The expression of mitochondrial genes is known to affect the quality, fertilization, and embryo development of oocytes [50]. Additioally, the expression of GDF-9 and BMP-15 is essential for the development and function of mouse [51] and human [52] oocytes, and supplementation of exogenous BMP-15 or GDF-9 during IVM significantly increased the development potential of oocytes in bovine [53] and in mouse [54] models. Thus, the expression levels of ATPase 6, ATPase 8, BMP-15, and GDF-9 mRNA are good indicators of the quality of oocytes. The results showed that, melatonin (10^−9^ M) significantly upregulated relative mRNA expressions of ATPase 6, BMP-15, and GDF-9 of IOs during their IVM (Figure 6), indicating that melatonin significantly improved the quality of oocytes. Moreover, the apoptosis of oocytes may result from a poor mitochondrial function and excess ROS under in vitro conditions [55]. The apoptosis was triggered by caspase-3, which is activated by cytochrome c release from mitochondria. Bcl-2 can interfere with cytochrome c release and, therefore, inhibit caspase-3 activation [56,57]. In the current study, melatonin downregulates caspase-3 while upregulating Bcl-2 (Figure 5), which is another mechanism by which melatonin improves the quality of IOs and promotes their maturation and embryo development. The detailed mechanisms are illustrated in the Figure 8.

## 4. Materials and Methods

### 4.1. Chemicals

NaCl, KCl, Na pyruvate, NaHCO_3_, hemicalcium l-lactate, bovine serum albumin (BSA), l-glutamine, essential amino acids (EAA), nonessential amino acids (NEAA), NaH_2_PO_4_·H_2_O, MgCl_2_·6H_2_O, glucose, and medium were purchased from Sigma-Aldrich (St. Louis, MO, USA).

### 4.2. Animal Studies

All experimental animal protocols were approved and performed in accordance with the requirements of the Institutional Animal Care and Use Committee at China Agricultural University. The protocol approving number is XK662.

### 4.3. Oocytes Collection, In Vitro Maturation, Fertilization, and In Vitro Embryo Development

Bovine ovaries were collected from the local abattoir and transported to the laboratory within 4 h. The cumulus oocyte complexes (COCs) were aspirated from follicles which were 3–8 mm in diameter using an 18 G needle attached to a 10 mL disposable syringe. Those with at least three layers of compact cumulus cells were used for IVM. Each group with 50 COCs were washed three times in 0.1% PVA–PBS and then cultured in maturation medium which contained different concentrations of melatonin in four-well dishes (Nunclon, Roskilde, Denmark) at 38.5 °C and 5% CO_2_.

IVM was performed for 22–24 h in 700 μL medium 199 (Gibco BRL, Carlsbad, CA, USA) supplemented with 10 μg/mL follicle stimulating hormone (FSH), 10 μg/mL luteinizing hormone (LH), 10% (*v*/*v*) fetal bovine serum (FBS, Hyclone; Gibco BRL, Grand Island, NY, USA), and 10 μg/mL estradiol (E2). Oocyte maturation was based on methods described in a previous study [20]. 

After maturation, oocytes were washed three times in Brackett and Oliphant (BO) wash medium [58], and aliquoted into groups of 15–20 oocytes and washed three times in BO fertilization medium consisting of 10 mM caffeine sodium benzoate and 0.5% fatty acid-free BSA, and then transferred into a 50 μL drop of fertilization medium in a Petri dish (Nunclon) and placed under mineral oil and 5% CO_2_ in humidified air at 38.5 °C. Frozen semen was thawed at 37 °C for 30 s. The sperm was washed three times by centrifugation at 1800 *g* for 5 min in 3 mL BO wash medium. Then, the sperm motility and concentration were determined. Sperm pellets were re-suspended in BO wash medium to a volume of 2 mL. Sperm suspension (50 μL) was added to each fertilization drop, giving a total concentration of 10 × 10^6^ spermatozoa/mL. Oocytes and sperm were incubated together under 5% CO_2_ in humidified air at 38.5 °C for 8 h before in vitro culture.

Presumptive zygotes were obtained after in vitro matured oocytes were fertilized in BO medium, and 15–20 zygotes were cultured in 60 μL CR1aa medium which supplemented with 3 mg/mL BSA (embryo culture tested fraction V, A-3311) for two days and 6% (*v*/*v*) fetal bovine serum (FBS) was added into the culture medium from day 3 until day 8 by changing half of the media every two days. The cleavage rate was recorded at 48 h, and blastocyst rate, hatched blastocyst rate, and mean cell number/blastocyst were observed on day 8.

### 4.4. Classification and Grouping of the Retrieved Oocytes

**Figure 9:**
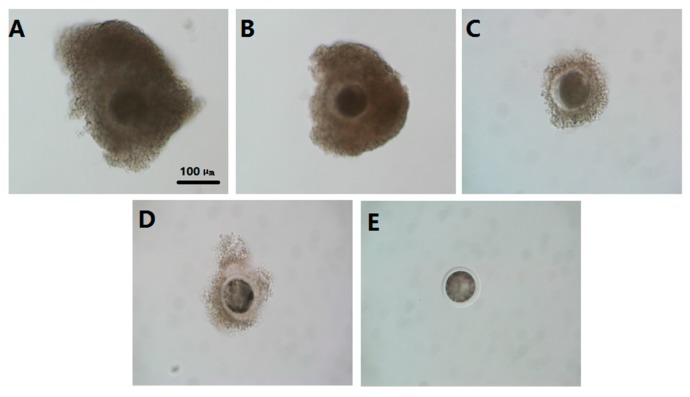
Classification criteria of GV oocytes. (**A**) COCs control, with an unexpanded cumulus mass having four or more layers of cumulus cells surrounding the zona pellucida and with homogenous cytoplasm; (**B**) COCs control with an unexpanded cumulus mass having three to four layers of cumulus cells and with relative homogenous cytoplasm; (**C**) oocytes with an unexpanded cumulus mass having under three layers of cumulus cells with regular cytoplasm or irregular shrunken cytoplasm; (**D**) oocytes partially denuded of cumulus cells and with irregular shrunken cytoplasm; and (**E**) oocytes completely denuded of cumulus cells and with irregular shrunken cytoplasm. C,D and E were classified as IOs. scale bar = 100 μm.

The collected oocytes were classified into four categories based on surrounding cumulus cell layers and homogeneity of ooplasm, as per the criterion established by methods described before [59] with some modifications: grade A (COCs control, with an unexpanded cumulus mass having four or more layers of cumulus cells surrounding the zona pellucida and with homogenous cytoplasm); grade B (COCs control with an unexpanded cumulus mass having three to four layers of cumulus cells and with relative homogenous cytoplasm); grade C (oocytes with an unexpanded cumulus mass having under three layers of cumulus cells with regular cytoplasm or irregular shrunken cytoplasm) and grade D + E (oocytes partially denuded or completely denuded of cumulus cells and with irregular shrunken cytoplasm) (Figure 9). The graded oocytes were further categorized into two groups: Group 1—good quality which included A and B grade COCs; and Group 2—inferior oocytes which included C and D + E grade oocytes IOs.

### 4.5. Assessment of Oocyte Maturation

The MII oocyte phase was determined by evaluating the presence of the first polar body, according to the method described by Zhao [60]. After 23 h IVM, IOs and COCs were denuded of cumulus cells by vortexing in 0.1% (*w*/*v*) hyaluronidase for 2–3 min. Then, they were fixed in methanol for 10 min, mounted on a slide, stained with 10 g/mL Hoechst 33342 and the presence or absence of polar bodies was determined by epifluorescence microscope (SP2; Leica, Wetzlar, Germany) (Figure 1B).

### 4.6. Measurement of Reactive Oxygen Species (ROS) and Glutathione (GSH)

The 2,7-dichlorodihydrofluorescein diacetate (H2DCFDA) (Beyotime, Jiangsu, China) and Cell Tracker Blue CMF2HC molecular probes (Invitrogen Inc, Carlsbad, CA, USA) were used to detect intracellular ROS and GSH levels, respectively. GV-stage oocytes were collected from bovine ovary and divided into three groups (IOs, IOs with 10^−9^ M melatonin-treated, COCs control) and cultured for 23 h in maturation medium. Then, MII-stage oocytes were collected and denuded from adherence of cumulus cells, then they were incubated (in the dark) for 30 min in M2 medium containing H2DCFDA (10 μM) or Cell Tracker Blue (10 μM), respectively. The MII-stage oocytes were collected and denuded from the adherence of cumulus cells, and placed in 30 μL M2 droplets, and then the fluorescence was observed using an epifluorescence microscope. The fluorescence intensity was analyzed using ImageJ software (version 1.40; National Institutes of Health, Bethesda, MD, USA).

### 4.7. Oocytes Mitochondrial Distribution Assay

MitoTracker Red CMRox (Life Technologies, Grand Island, NY, USA) was used to detect mitochondrial distribution. GV-stage oocytes were collected and divided into three groups (IOs, IOs + MT (10^−9^ M), COCs control, respectively). Cells were cultured in maturation medium for 23 h. Then, MII-stage oocytes were collected and denuded from the adherence of cumulus cells and they were incubated in PBS medium supplemented with 100 nM dye at 37 °C for 40 min. Oocytes were then washed and analyzed by epifluorescence microscope (TE300; Nikon, Tokyo, Japan). Oocytes with a uniform granulated distribution of active mitochondria were scored as granulated distribution (GD), and the oocytes with a massive clustering distribution of mitochondria were scored as massive distribution (MD). The images were observed and scored by three independent persons who were unaware of this study.

### 4.8. Detection of ATP Levels in Oocytes

MII-stage oocytes were washed with PBS-PVA collected for ATP measurement, ATP levels were determined using a commercially-available adenosine 5′-triphosphate (ATP) bioluminescent somatic cell assay kit (FLASC, Sigma-Aldrich, St. Louis, MO, USA) according to the manufacturer’s instructions. Briefly, oocytes were transferred into a 96-well plate with 45.8 μL ATP assay buffer, then 0.2 μL ATP probe, 2 μL ATP converter, and 2 μL developer mix were added. The plate was placed at room temperature for 30 min. ATP levels were measured using a luminometer (Bioluminat Junior, Berthold, Germany).

### 4.9. Assessment of Embryo Quality

The quality of blastocysts was assessed by Hoechst 33342 staining for 10 min. After rinsing in 0.1% PVA-PBS medium, blastocysts were mounted on a clean glass slide, then covered with a coverslip and examined under an inverted microscope (TE300; Nikon, Tokyo, Japan) equipped with epifluorescence.

### 4.10. RNA Isolation and Reverse Transcriptional PCR

Fresh immature oocytes were subjected to denuding by pipetting in 0.1% hyaluronidase enzyme in 0.1% PVA-PBS, whereas matured ones were denuded by pipetting in 0.1% PVA-PBS only. Denuded oocytes and cumulus cells were washed twice in D-PBS solution. Both embryo collection and RT-PCR procedures were performed according to the instructions on the Cells-to-cDNATM Kit (Ambion Company, Grand Island, NY, USA; AM1722). Before the final step of gene expression analysis, each cDNA sample was first amplified with a pair of primers specific for bovine β-actin mRNA (Table 4) to screen the samples for contamination with genomic DNA. The PCR was run as follows: initial denaturation at 95 °C for 5 min, denaturation at 94 °C for 30 s, annealing at Tm (°C) for 45 s, and then extension at 72 °C for 30 s. The above procedures were repeated for 35 cycles with a final extension at 72 °C for 5 min.

### 4.11. Detection of HSP90 in Oocyte via Immunofluorescence

Bovine oocytes were fixed with 4.0% neutral-buffered paraformaldehyde containing 0.3% Triton X-100 at 37 °C for 45 min; nonspecific binding was blocked using PBS supplemented with 0.5% BSA, 0.1% Triton X-100, and 5% fatal bovine serum (FBS) at 37 °C for 1 h, and then antibody of HSP90 (final concentration 1:200, Abcam, Anti-Hsp90, [AC88] ab13492) was added to PBS containing 0.5% BSA, and 5% FBS, and incubated at 4 °C overnight. Oocytes were then washed three times with PBS containing 0.5% BSA and 0.01% Triton X-100 (15 min per wash) and incubated with goat anti-mouse IgG-FITC antibody (1:100 dilution, Santa Cruz Bio Inc., Santa Cruz, CA, USA) at 37 °C for 1 h. After washing, the cell nucleus was counterstained with Hoechst 33342 and analyzed by epifluorescence microscope (SP2; Leica, Wetzlar, Germany). Fluorescence intensity was analyzed using ImageJ software (version 1.40; National Institutes of Health, Bethesda, MD, USA).

### 4.12. Statistical Analysis

The data are expressed as the mean values ± standard error of the mean (SEM). The data were analyzed using univariate analysis of variance (ANOVA) followed by Duncan's test using SPSS 18.0 statistical software (SPSS Inc., Chicago, IL, USA). The significant difference was set up when the *p* < 0.05.

## 5. Conclusions

In conclusion, melatonin at the 10^−9^ M which is the physiological concentration promoted the maturation rate of bovine IOs, and increased their cleavage, blastocyst, and hatched blastocyst rates of IVF embryos. A novel mechanism has been observed, that is, melatonin improves to the normal distribution of mitochondria and preserves the ATP production in IOs. Melatonin exhibited its anti-oxidative and anti-apoptotic activities and upregulated several gene expressions which are related to the oocytes’ maturation and embryo development. All these lead to the improvement of the quality of IOs. The mechanisms are summarized in the Figure 9. As a result, it appeared that melatonin treatment can serve as an effective method to improve the quality of IOs and this would have important biological and commercial values in in vitro embryo production. The discoveries also provide an important reference for application of melatonin in human test-tube baby technology.

## Figures and Tables

**Table 1:** The effect of melatonin on the nuclear maturation of bovine oocytes.

Groups	No. of Oocytes Observed	No. of MII Oocytes (%)
IOs	293	174 (59.4 ± 3.14) ^Aa^
IOs + MT	311	222 (71.4 ± 1.88) ^Ab^
COCs	421	370 (87.9 ± 0.64) ^Bc^

^a,b^^,c^ Values with different superscripts represent significant difference within the same column (*p* < 0.05); ^A,B^ Values with different superscripts represent highly significant difference within the same column (*p* < 0.01), IOs, the inferior bovine oocytes ; MT, melatonin; COCs, cumulus–oocyte complexes.

**Table 2:** The effect of melatonin on IVF Embryo Developmental potential of oocytes.

Groups	No. of MII Oocytes	No. of Cleavage Embryos (%)	No. of Blastocysts (%)	No. of D8 Hatched Blastocysts (%)
IOs	215	142 (66.15 ± 2.64) ^Aa^	47 (33.1 ± 0.87) ^Aa^	3 (6.4 ± 2.36) ^Aa^
IOs + MT	228	182 (79.8 ± 2.42) ^ABb^	70 (38.5 ± 1.11) ^ABb^	9 (12.9 ± 2.99) ^ABa^
COCs	369	334 (90.5 ± 0.60) ^Bc^	147 (44.0 ± 0.74) ^Bc^	42 (28.6 ± 1.01) ^Bb^

^a,b^^,c^ Values with different superscripts represent significant difference within the same column (*p* < 0.05); ^A,B^ Values with different superscripts represent highly significant difference within the same column (*p* < 0.01), IOs, inferior bovine oocytes; MT, melatonin; COCs, cumulus–oocyte complexes.

**Table 3:** The effect of melatonin on Blastocyst cell number of IVF embryos using inferior oocytes.

Groups	Cell Number/Blastocyst	Pooled SEM
IOs	104.9 ^Aa^	3.51
IOs + MT	115.8 ^ABb^	1.71
COCs	122.3 ^Bb^	3.57

^a,b^ Values with different superscripts represent significant difference within the same column (*p* < 0.05); ^A,B^ Values with different superscripts represent highly significant difference within the same column (*p* < 0.01), IOs, inferior bovine oocytes; MT, melatonin; COCs, cumulus-oocyte complexes.

**Table 4:** Primers used in this study.

Genes	Primer Sequence(5′-3′)	T_m_ (°C)	Product Size (bp)
β-Actin	Forward:TGACGTTGACATCCGTAAAGACC	60	117
	Reverse: GTGCTAGGAGCCAGGGCAG		
Gpx-4	Forward: TGTGCTCGCTCCATGCACGA	60	224
	Reverse: CCTGGCTCCTGCCTCCCAA		
SOD1	Forward: GCTGTACCAGTGCAGGTCCTCA	60	228
	Reverse: CATTTCCACCTCTGCCCAAGTC		
Caspase-3	Forward: CAGACAGTGGTGCTGAGGATGA	60	211
	Reverse: GCTACCTTTCGGTTAACCCGA		
Bcl-2	Forward: GACTGACACTGAGTTTGGCTACG	60	152
	Reverse: GAGTCCTTTCCACTTCGTCCTG		
Bax	Forward: GGCTGGACATTGGACTTCCTTC	60	161
	Reverse:TGGTCACTGTCTGCCATGTGG		
BMP-15	Forward: GAGGCTCCTGGCACATACAGAC	60	134
	Reverse:CTCCACATGGCAGGAGAGGT		
GDF-9	Forward: CAGAAGCCACCTCTACAACACTG	60	95
	Reverse: CTGATGGAAGGGTTCCTGCTG		
ATPase6	Forward: GAACACCCACTCCACTAATCCCAAT	60	147
	Reverse: GTGCAAGTGTAGCTCCTCCGATT		
ATPase8	Forward: CACAATCCAGAACTGACACCAACAA	60	129
	Reverse: CGATAAGGGTTACGAGAGGGAGAC		
HSP90	Forward: TCATTGGCTATCCCATCACTCT	60	324
	Reverse: AATCGTTGGTCAGGCTCTTGTA		
